# Synthesis of Silica-Coated Fe_3_O_4_ Nanoparticles by Microemulsion Method: Characterization and Evaluation of Antimicrobial Activity

**DOI:** 10.1155/2020/4783612

**Published:** 2020-04-12

**Authors:** Goshu Asab, Enyew Amare Zereffa, Teshome Abdo Seghne

**Affiliations:** Department of Applied Chemistry, School of Applied Natural Science, Adama Science and Technology University, P.O. Box 1888, Adama, Ethiopia

## Abstract

Magnetite and silica-coated magnetite (Fe_3_O_4_) nanoparticles (NPs) were synthesized by water-in-oil (W/O) microemulsion method from hydrated ferric nitrate, ferrous sulfate precursors and ammonia a precipitating agent with the assistance of Tween-80 and SDS surfactants. The synthesized materials were characterized by X-ray diffraction, scanning electron microscopy, thermal analyzer, and infrared spectroscopy. X-ray diffraction pattern of Fe_3_O_4_ showed that particles were phase pure with a cubic inverse spinel structure and FT-infrared spectra confirmed the presence of Fe-O bond in tetrahedral and octahedral interstitial sites. The crystallite size determined from powder XRD data with Scherer's equation was in the range of 7.3 ± 0.05 nm–10.83 ± 0.02 nm for uncoated Fe_3_O_4_ and 16 ± 0.14 nm for silica-coated Fe_3_O_4_ NPs. The SEM micrographs of the uncoated Fe_3_O_4_ oxide revealed the agglomeration of the magnetite (Fe_3_O_4_) particles. But the silica-coated Fe_3_O_4_ oxide exhibited homogeneous distribution of particles with relatively less agglomerate of the particles. The particle size of Fe_3_O_4_ NPs slightly increased with the temperature and precursor concentration. The antimicrobial activities of Fe_3_O_4_ and silica-coated Fe_3_O_4_ nanoparticles were tested against Gram-negative (*Escherichia coli* and *Pseudomonas aeruginosa*) and Gram-positive (*Staphylococcus aureus* and *Bacillus subtilis*) bacteria. Both Fe_3_O_4_ and silica-coated Fe_3_O_4_ NPs demonstrated better antimicrobial activities.

## 1. Introduction

Nanoscience and nanotechnologies are attracting the interest of researchers and technologists in several fields for the development of nanoscale materials and devices with new properties and functions. Nanotechnology is the study of manipulating matter on an atomic and molecular scale [1]. Our present environments are filled with various types of pollutants emitted from processes. Nanotechnology is playing an important role in providing effective solutions to the diverse environmental challenges [2]. Nanoparticles are particles between 1 and 100 nanometers (nm) in size with a surrounding interfacial layer [3]. Due to their size, nanoparticles demonstrate unique and controllable properties that are different from the macroscopic scale [4].

Iron oxides are the most important transition metal oxides with different technological significance. Iron oxides are found in nature in different forms. Magnetite (Fe_3_O_4_), maghemite (ϒ-Fe_2_O_3_), and hematite, *α*-(Fe_2_O_3_) are the most common oxides of iron [5]. Among all iron oxides, magnetite Fe_3_O_4_ possesses the most interesting properties due to the presence of iron cations in two valence states, Fe^3+^ and Fe^2+^, in the inverse spinel structure. The cubic spinel Fe_3_O_4_ is ferromagnetic at a temperature below 858 K [6].

However, magnetite nanoparticles suffer from two major issues such as rapid agglomeration and oxidation by oxygen of the air. The coating is the most common surface modification approach to conjugate the organic or inorganic materials onto the surface of iron oxide nanoparticles (IONPs). This method is not only preventing the oxidation and agglomeration of IONPs but it also provides the possibility for further functionalization [7].

Recently, a wide range of techniques has been developed for the preparation of nanomaterials. These techniques include physical methods such as mechanical milling [8] and inert gas condensation. In addition, chemical methods such as chemical reduction, photochemical reduction, electrodeposition, hydrothermal, sol-gel, and microemulsion synthesis are also available [9].

The synthesis of Fe_3_O_4_ MNPs through modified coprecipitation by using sodium citrate under argon gas for ferrofluid applications was reported by Hong et al., 2009 [10]. Magnetic nanoparticles, with mean size between 5 and 10 nm, were produced by thermal decomposition of iron (III) chloride hexahydrate (FeCl_3_-6H_2_O) in 2-pyrrolidone and successively dispersed in water and polyethylene glycol 400 [11]. Nanosized magnetic particles with average sizes from 4 to 12 nm and standard deviation ranging from 0.2 to 0.3 were prepared using microemulsions [12].

Magnetite nanoparticles around 4 nm in diameter have been prepared by the controlled hydrolysis with ammonium hydroxide of FeCl_2_ and FeCl_3_ aqueous solutions within the reverse micelle nanocavities generated by using AOT as surfactant and heptanes as the continuous oil phase [13]. Magnetite (Fe_3_O_4_) nanoparticles (MNPs) and silica-coated magnetite nanoparticles (SMNPs) were synthesized as adsorbents for removing humic acid (HA) from water resources by modified coprecipitation technique [14]. Today, there are over hundreds of ongoing clinical trials involving nanoparticles to treat disease. According to the report of [15], 76% of the publications and 59% of the patents are the market sector that dominates the nanomedicine part. The preparation of magnetic nanoparticles for biomedicine applications by different methods was thoroughly reviewed by [16]. The group also addressed some relevant findings and synthetic routes to produce magnetic nanoparticles. Magnetite nanoparticles were also fabricated by spark erosion, electric explosion of wire, and Infrared Pulsed Laser Ablation [17–19].

Fe_3_O_4_-NPs-based biomedical applications have received considerable attention due to their diverse methods of synthesis, biocompatibility, and environmental safety. Therefore, Fe_3_O_4_ NPs may be reasonable candidates for their potential use as antibacterial therapy. The aim of the present study is to synthesize and characterize Fe_3_O_4_ and silica-coated Fe_3_O_4_ nanoparticles by a microemulsion method and evaluate their antimicrobial activity.

## 2. Experimental

### 2.1. Materials

The chemicals used for the synthesis of Fe_3_O_4_ NPs and silica-coated Fe_3_O_4_ NPs are iron (III) nitrate nonahydrate (Fe(NO_3_)_3_·9H_2_O, 99% Sigma Aldrich), iron (II) sulfate heptahydrate (FeSO_4_·7H_2_O, 99·5% Sigma Aldrich), ammonia (25% NH_3,_ ultra-pure, France, Carlo Erba), polyoxyethylene sorbitan monooleate (Tween-80), sodium dodecyl sulfate (SDS), 1-butanol (CH_3_ (CH_2_)_3_OH, 99.5%, Ranchem India), n-heptanes (C_7_H_16_, 99%, Ranchem India), silicon oxide (SiO_2_), acetone, and distilled water. All the chemicals are analytical grade and used without further purifications. We have also used Gram-negative (*Escherichia coli* and *Pseudomonas aeruginosa*) and Gram-positive (*Staphylococcus aureus* and *Bacillus subtilis*) bacteria as well as fungi (*C. albicans*).

### 2.2. Synthesis of Fe_3_O_4_ and Silica-Coated Fe_3_O_4_ Nanoparticles

The Fe_3_O_4_ magnetic NPs were prepared by the water-in-oil microemulsion method (W/O) with slight modifications of earlier reported method [20, 21]. The microemulsion system used in this study consisted of Tween-80 as the surfactant, 1-butanol as the cosurfactant, n-heptanes as the continuous oil phase, and an aqueous solution of reactants as the dispersed phase. The ratio of surfactant to cosurfactant was fixed at 1 : 1 on the volume basis, i.e., 20 mL of tween-80/butan-1-ol and 60 ml n-heptane.

**Figure 1:**
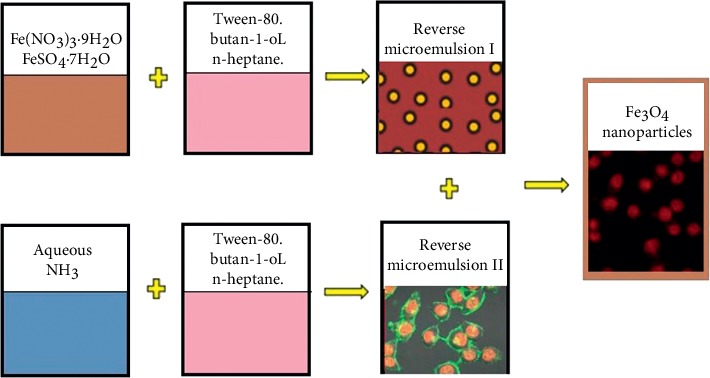
Synthesis procedure of Fe_3_O_4_ nanoparticles by microemulsion (W/O) method.

The precursor solution (solution I) contains 2 : 1 mole ratio of iron salts, Fe(NO_3_)_3_·9H_2_O (20 mL of 0.4 M), and FeSO_4_·7H_2_O (20 mL of 0.2 M) dissolved in 80 mL of a mixture of Tween-80/butan-1-ol/n-heptane. This mixture results in the formation of a reverse microemulsion. Solution II contains 80 mL of Tween-80/butan-1-ol/n-heptanes and 50 mL of 25% aqueous NH_3_. These solutions were stirred at the rate of 300 rpm for 30 minutes at room temperature. Solution II was added to solution I and the combined mixture was stirred continuously with a speed of 1000 rpm for 150 minutes at different temperatures of 30, 50, and 80°C, and the precipitate was washed several times by distilled water and acetone to eliminate the ammonia and the surfactant. Finally, the magnetic Fe_3_O_4_ nanoparticles were obtained after drying in a vacuum oven and recorded as Fe_3_O_4_-30T, Fe_3_O_4_-50T, and Fe_3_O_4_-80T, respectively, and kept for characterization and further use (see Figure 1).

The second batch of magnetite was synthesized from the same precursors by replacing surfactant Tween-80 with SDS; the same amount and type of precursors, oil phase, and base were used. The third batch of magnetites was synthesized from the same precursors with different concentrations to investigate the precursor's concentration effect on crystallites size with slight modification [22]. Finally, silica-coated Fe_3_O_4_ (Fe_3_O_4_@SiO_2_) nanoparticles were prepared in a similar manner with batch one with the addition of 10 mL of 0.2 M SiO_2_ aqueous solution in solution II and the reaction is carried out at optimized temperature.

### 2.3. Methods of Antimicrobial Evaluation

#### 2.3.1. Agar Well Diffusion Method

Antimicrobial testing was performed against Gram-negative (*Escherichia coli* and *Pseudomonas aeruginosa*) and Gram-positive (*Staphylococcus aureus* and *Bacillus subtilis*) bacteria and *Candida albicans* fungi. Microbial strains were obtained from the pastor institute. Microbial cultures were maintained on nutrient Muller-Hinton agar at 37°C, and the cultures were kept in appropriate media slants and stored at 4°C until used. The antibiotic gentamicin was used as a positive control and DMSO as a negative control in this study. The antimicrobial activity of the different concentration of Fe_3_O_4_ and silica-coated Fe_3_O_4_ NPs was evaluated by agar well disc diffusion method adopted from [23] with some modification. Sterile nutrient plates were prepared. The plates were allowed to solidify for 5 minutes and wells of 6 mm were punctured in selected areas on different plates using a good borer. 1 mL inoculum suspension of Gram-negative (*Escherichia coli* and *Pseudomonas aeruginosa*) and Gram-positive (*Staphylococcus aureus* and *Bacillus subtilis*) bacteria and *C. albicans* was swabbed uniformly over the surface of the agar plate. 100 mg uncoated Fe_3_O_4_ and 150 mg of silica-coated Fe_3_O_4_ nanoparticles were dissolved in 10 mL and 15 mL DMSO, respectively, to obtain 10 mg/mL and 15 mg/mL of solutions. Then, 100 *µ*L of each prepared NPs was loaded into the well, and the plates were kept for incubation at 37°C for 24 hours. The antimicrobial activity was evaluated in terms of zone of inhibition and measured and recorded in millimeters using a ruler. Clear inhibition zones formed around the well indicated the presence of antimicrobial activity.

### 2.4. Characterization Methods

Thermal gravimetric analysis (TGA) was carried out using a simultaneous DTA-TG apparatus (DTG-60H, Shimadzu Co., Japan) to determine the thermal stability of the synthesized material. X-ray diffraction patterns of the synthesized NPs were recorded using a BRUKER D8 Advance X-ray diffractometer equipped with a Cu target for generating a Cu K*α* radiation (wavelength 1.5406 Å) as the X-ray source. The measurements were made at room temperature, and the accelerating voltage and the applied current were 40 kV and 30 mA, respectively. The instrument was operated under step scan type with step time and degree (2*θ*) of 0.4 s and 0.020°, respectively, over 10° to 80°, to investigate the phase formation of the sample. The crystallite size of the NPs was determined from the XRD pattern by using Debye-Scherer's equation.

FTIR spectra were recorded in the solid phase using the KBr pellet technique in the regions of 4000–400 cm^−1^. FTIR spectra yield information on the chemical bonds between the Fe_3_O_4_ core and the organic surface coverage. Scanning electron microscopy (SEM) was used to determine the morphology of the synthesized magnetite NPs. A nanoparticle size analyzer was used to perform dynamic light scattering analysis (DLS, Brookhaven Instrument Corporation) with ZetaPALS particle sizing software version 5.23, to determine particle size distributions.

## 3. Results and Discussion

### 3.1. Characterization of Fe_3_O_4_ and Silica-Coated Fe_3_O_4_ NPs

#### 3.1.1. Thermogravimetric Differential Thermal Analysis

**Figure 2:**
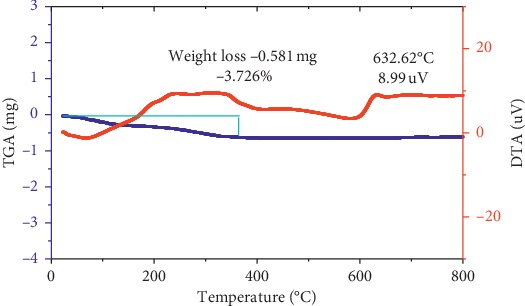
TG-DTA curves for Fe_3_O_4_ nanoparticles using the Tween-80 surfactant at 30°C.

Thermogravimetric analysis (TGA) and differential thermal analysis (DTA) of Fe_3_O_4_ synthesized with Tween-80 surfactant at 30°C are presented in Figure 2. The TGA curve shows a mass loss of the sample whereas the DTA curve indicates the energy gain or loss during the process. The Fe_3_O_4_ nanoparticles were thermally stable, and there was no essential weight loss over the entire temperature range in the TG curve (Figure 2).

The total weight loss as shown in TG curve exhibited only 3.726% of weight loss, where the largest portion of this weight loss occurred at the temperature of 25–250°C which could be attributed to the removal of the physically adsorbed water and/or hydroxyl groups on the surface of Fe_3_O_4_ nanoparticles. The thermal result implies that the surfactant and cosurfactants were removed through washing from the as-synthesized Fe_3_O_4_ NPs and thermal treatment was not necessary for their removal.

#### 3.1.2. X-Ray Diffraction

**Figure 3:**
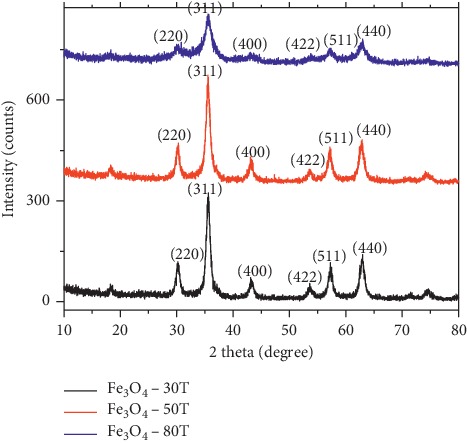
XRD patterns of Fe_3_O_4_ nanoparticles using the Tween-80 surfactant at different temperatures.

Figure 3 illustrates the XRD patterns of Fe_3_O_4_ synthesized with the Tween-80 surfactant at different temperatures (30°C, 50°C, and 80°C). The powder diffraction patterns showed major peaks at 2*θ* values of 30.2°, 35.6°, 43.2°, 53.56, 57.2°, and 62.8° from the reflection crystal planes (220), (311), (400), (422) (511), and (440), respectively. The position and relative intensity of all diffraction peaks match well with those of the magnetite (JCPDS Card. No. 79-0418), and the narrow sharp peaks of materials indicate that the nanoparticles have relatively high crystallinity without the appearance of the impurities such as goethite *α*-FeO(OH) and hematite (Fe_2_O_3_) corresponding to the diffraction peaks of (110) and (104) at 2*θ* positions of 21.22° and 33.15. The particle size was determined by taking the average sizes of the peaks *D*_220_, *D*_311_, *D*_400_, *D*_511_, and *D*_440_.

The calculated mean crystallite size of the Fe_3_O_4_ nanoparticles at three different temperatures 30°C, 50°C, and 80°C were found to be 7.85 ± 0.01 nm, 8.41 ± 0.13 nm, and 10.83 ± 0.02 nm, respectively. The lattice parameter “*a*” and interplanar spacing d_hkl_ were determined by Bragg's equation. The crystal structure of the nano-Fe_3_O_4_ particles belongs to a cubic system with lattice parameters (*a* = 8.354, 8.366, and 8.356 Å) at three different temperatures with d-spacing 2.5189, 2.5226, and 2.5199, respectively, for the miller index of major peak *D*_311_. The lattice parameter and particle size of Fe_3_O_4_ NPs synthesized at different temperatures were found to be comparable. As a result, room temperature synthesis of the material is possible by the method selected.

**Figure 4:**
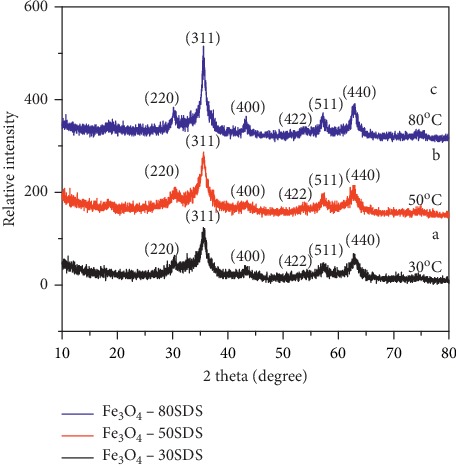
XRD patterns of Fe_3_O_4_ nanoparticles using SDS surfactant at temperatures 30°C, 50°C, and 80°C.

Diffraction patterns of the Fe_3_O_4_ synthesized using SDS (Figure 4) at different temperatures (30°C, 50°C, and 80°C) are 2*θ* = 30.3°, 35.58°, 43.33°, 53.6°, 57.34°, and 62.86° corresponding to the miller indexes (220), (311), (400), (422), (511), and (440), respectively, which are the characteristic peaks of the Fe_3_O_4_ crystal with a cubic spinel structure. It is clear that the phase of the XRD pattern matches with (JCPDS Card No. 79-0418) file. The calculated mean crystallite size of the Fe_3_O_4_ NPs synthesized by using SDS surfactant at different temperatures were found to be 8.07 ± 0.21 nm, 8.1 ± 0.04 nm, and 9.44 ± 0.02 nm, respectively.

The sizes of NPs produced by both surfactants were found to slightly increase with rise in temperature which might be due to agglomeration kinetics. The reaction temperature change for such a method does not favor large particle formation. Temperature influences strongly the nucleation and growth mechanisms [24]. When the temperature increases, the particle size becomes bigger and the particle size distribution is irregular. The increase in frequencies of the collision between the particles leads to the kinetic energy of collision increasing; this makes the nanoparticles have a strong tendency to overcome the potential barrier between them and agglomerate into large particles.

**Figure 5:**
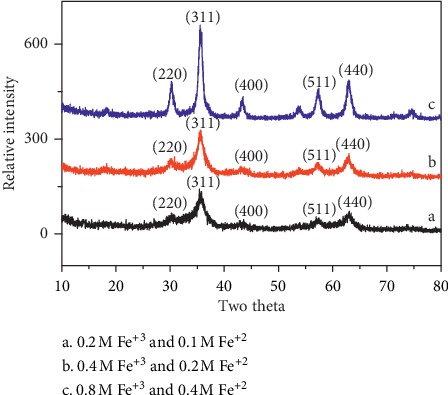
XRD patterns of Fe_3_O_4_ nanoparticles at 30°C by varying precursor concentration.

*Effect of the Concentration of Fe* ^*3+*^ *and Fe* ^*2+*^. Fe_3_O_4_ NPs were prepared with different concentrations of Fe^2+^ and Fe^3+^ in the aqueous phase while all other parameters were kept constant to investigate the effect of concentration of the precursor. The concentration of the precursor has a major influence on the size of nanoparticles, with high concentration; larger nanoparticles were formed [25].  Figure 5 shows the XRD pattern of Fe_3_O_4_ nanoparticles at various precursor concentrations. The crystallite size of nanoparticles was found to be 7.73 ± 0.05 nm, 8.64 ± 0.03, and 10.3 ± 0.02 nm from the X-ray line broadening.

As the concentration of precursor changes from 0.4 M of Fe^+3^ and 0.2 M of Fe^+2^ to 0.2 M of Fe^+3^ and 0.1 M of Fe^+2^, the size of Fe_3_O_4_ NPs was found to decrease from 7.85 ± 0.01 nm to 7.3 ± 0.05 nm. Similarly, as the concentration of precursor changes from 0.4 M of Fe^+3^ and 0.2 M of Fe^+2^ to 0.8 M of Fe^+3^ and 0.4 M of Fe^+2^, the size of Fe_3_O_4_ NPs was found to increase from 7.85 ± 0.01 nm to 8.64 ± 0.03 nm. The size of nanoparticles was found to increase linearly with precursor concentration.

**Figure 6:**
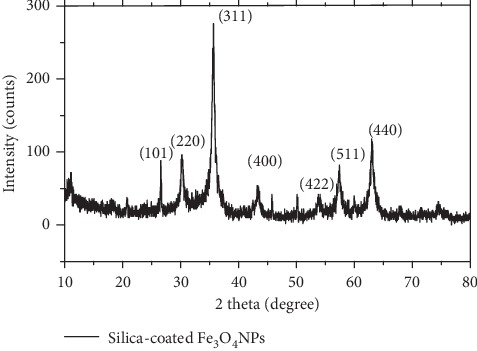
XRD pattern of silica-coated Fe_3_O_4_ NPs.

The XRD pattern of silica-coated Fe_3_O_4_ magnetic nanoparticles (Figure 6) exhibits diffraction patterns similar to that of Fe_3_O_4_ NPs. The diffraction peaks at 30.2°, 35.6°, 43.3°, 53.8°, 57.3°, and 63° refer to (220), (311), (400), (422), (511), and (440) planes of cubic inverse spinel Fe_3_O_4_, respectively. The additional peak at 26.6° and 51.1° degree corresponds to SiO_2_ (Yunusa, Ahmed, Bawa, Iyun, and Dauda, 2016), and the rest of peaks are similar to those found in XRD patterns of Fe_3_O_4_. The average crystal size of silica-coated Fe_3_O_4_ obtained by Scherer's formula was about 16 ± 0.14 nm using the peaks at *D*_220_, *D*_311_, *D*_400_, *D*_511_, and *D*_440_.

The silica-coated Fe_3_O_4_ NPs have a greater crystallite size than bare Fe_3_O_4_ nanoparticles. The increase in crystallite size may be due to the addition of a large amount of SiO_2_ in solution mixture and resulted in the expansion of Micelles (nanoreactors volume) in which the Fe_3_O_4_ crystal grows. A similar result was reported by Sachnin A. Kulkarni where the particle size increased with the increase in tetraethyl orthosilicate content.

#### 3.1.3. FTIR Spectral Analysis

**Figure 7:**
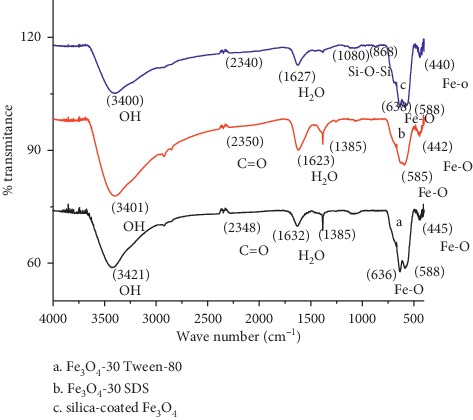
FTIR spectra of Fe_3_O_4_ NPs using (a) Tween-80 surfactant, (b) SDS surfactant, and (c) silica-coated Fe3O4 NPs.

Figure 7 demonstrates the FTIR spectra of Fe3O4 NPs synthesized with Tween-80, SDS surfactants, and silica-coated Fe3O4 NPs. The inverse spinel-type structure of Fe_3_O_4_ again was confirmed by IR bands, indicating the vibrations M_t_–O–M_O_ (*ν*_1_ ≈ 600–550 cm^−1^) and M_O_–O (*ν*_2_ ≈ 440–470 cm^−1^), where *M*_t_ and *M*_o_ correspond to the metal occupying tetrahedral and octahedral positions, respectively [26]. In Figure 7(a), the peaks at 3421 cm^−1^ and 2348 cm^−1^ indicate the presence of OH and C=O, respectively, probably due to atmospheric moisture and CO_2_, respectively. The presence of two strong absorption bands at around 636 and 588 cm^−1^ shows the formation of magnetic nanoparticles. Moreover, the band at 588 cm^−1^confirms Fe-O stretching vibration of tetrahedral sites of spinel structure and the absorption bands at 445 cm^−1^ can be attributed to tetrahedral and octahedral sites [27].

FTIR spectrum shows less intense H-O-H bending vibration in the region 1623–1089 cm^−1^, typical of the H_2_O molecule. These peak values nearly match with the reported values of [28]. A typical FTIR spectrum of the pure Fe_3_O_4_ nanoparticles synthesized using the SDS surfactant is shown in Figure 7(b). Two absorption bands at 585 and 442 cm^−1^ corresponding to the Fe–O bonds in tetrahedral and octahedral sites confirm the spinel-type structure of pure Fe_3_O_4_ nanoparticles [29]. The FTIR spectra of pure Fe_3_O_4_ nanoparticles also exhibit absorption bands appearing at 1623 cm^−1^, which can be attributed to hydroxyl groups that cover the surface of Fe_3_O_4_ nanoparticles due to aqueous media synthesis. The peak found at 3401 cm^−1^ was a characteristic of the stretching vibration of OH.

Figure 7(c), with an absorption peak at 588–638 cm^−1^, confirms the presence of a Fe–O bond related to the magnetite phase of magnetite nanoparticles. Bands at 868 cm^−1^ and 1080 cm^−1^ were due to symmetric and asymmetric linear vibrations of Si–O–Si, indicative of the formation of a silica shell with SiO_2_-modified magnetite [30]. This data supports the formation of SiO_2_ shell on Fe_3_O_4_ core. The transmittance of coated Fe_3_O_4_ NPs was slightly lower than that of Fe_3_O_4_ NPs because of the coating.

#### 3.1.4. Surface Morphology Analysis (SEM)

**Figure 8:**
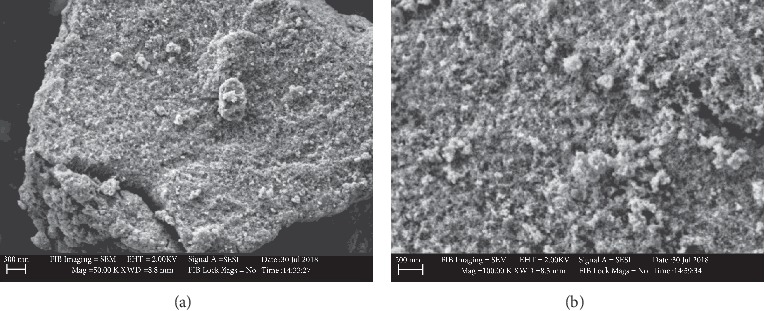
SEM micrographs of Fe_3_O_4_ NPs using Tween-80 surfactant at different magnifications (a) and (b).

SEM micrographs revealed the morphology of bare Fe_3_O_4_ nanoparticles and silica-coated Fe_3_O_4_ NPs. The micrographs were recorded at 2.00 kV of accelerating voltage using an 8.3 mm working distance at different magnifications by a high-resolution field emission scanning electron microscope (FE SEM). The SEM micrographs of Fe_3_O_4_ particles synthesized using the Tween-80 surfactant are shown in Figures 8(a) and 8(b).

The particles are homogeneously distributed with agglomerates and their size distribution of particles was in the range of 17.69 to 33.36 nm with a mean of 24 nm and a standard deviation of 0.03. Flower-like shapes of particles were observed at higher magnification (Figure 8(b)).

**Figure 9:**
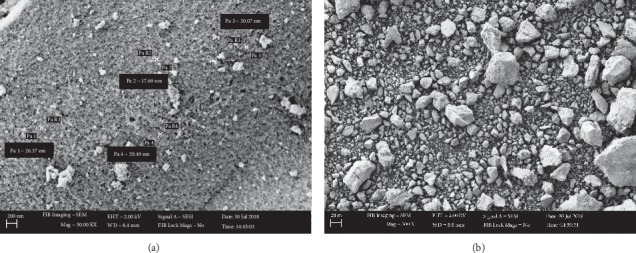
SEM micrographs of Fe_3_O_4_ NPs using SDS surfactant at different magnifications (a) and (b).

The SEM micrographs of Fe_3_O_4_ particles synthesized using the SDS surfactant revealed the presence of heterogeneous particle size distributions (Figures 9(a) and 9(b)). The size distribution of particles was found to be in the range between 17.69 and 30.07 nm. The mean diameter of the nanoparticles was found to be 25.9 nm with a standard deviation of 0.14.

**Figure 10:**
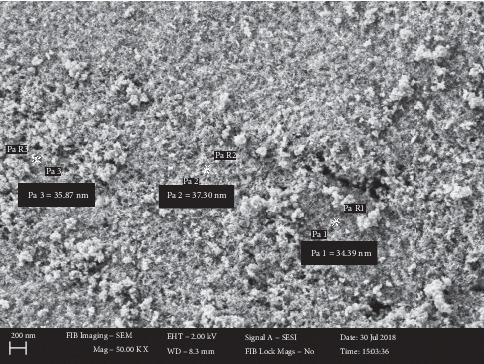
SEM micrograph of silica-coated Fe_3_O_4_ NPs.

Figure 10 shows the SEM image of the silica-coated magnetite. It showed that the particles were homogeneously distributed without any substantial agglomeration. Coating the surface of magnetite particles with suitable and nontoxic compounds has been proven to be one of the most efficient ways for providing stability of the nanoparticles. On the other hand, agglomeration of coated Fe_3_O_4_ NPs was reduced due to surface modification [31]. The size distributions of particles were in the range of 34.39 to 37.3 nm which exhibit a relatively narrow size distribution. From the micrographs, the mean diameter of silica-coated Fe_3_O_4_ nanoparticles was calculated to be 35.3 nm and a standard deviation of 0.04.

#### 3.1.5. Particle Size Analysis (DLS)

**Figure 11:**
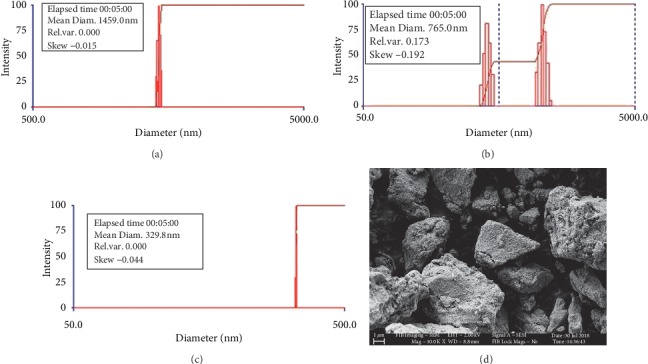
Particle size analysis. (a) Fe_3_O_4_ NPs synthesized using the Tween-80 surfactant at 30°C. (b) Silica-coated Fe_3_O_4_ NPs synthesized using the Tween-80 surfactant at 30°C. (c) Fe_3_O_4_ NPs synthesized using SDS surfactant at 30°C. (d) SEM micrographs of Fe_3_O_4_ synthesized using the Tween-80 surfactant after the DLS analysis.

**Figure 12:**
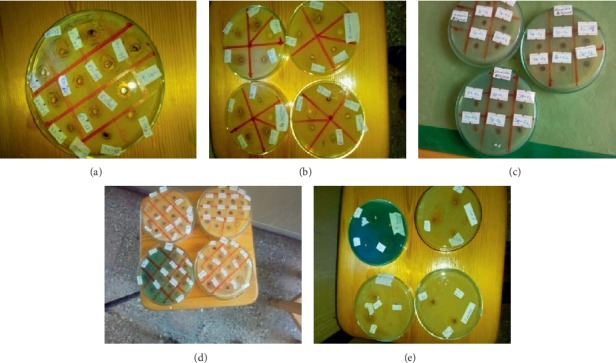
Antimicrobial activities of various Fe_3_O_4_ NPs samples and silica-coated Fe_3_O_4_ NPs on four bacteria strains, (a) *Staphylococcus aurous* (ATCC 25923), (b) *Bacillus subtilis* (ATCC6633), (c) *Escherichia coli* (ATCC25922), and (d) *Pseudomonas aeruginosa* (ATCC7553), and (e) *Candida albicans* fungi.

Fe_3_O_4_ NPs synthesized using the Tween-80 surfactant at 30°C exhibited a mean diameter of 1459.8 nm as shown in Figure 11(a) confirming the effect of *crystal* sizes on the *agglomeration* of Fe_3_O_4_ NPs. Fe_3_O_4_ NPs synthesized using SDS surfactant at 30°C exhibited a mean diameter of 765 nm as shown in Figure 11(b). Silica-coated Fe_3_O_4_ NPs synthesized under similar conditions are found to have a particle size of 329.8 nm. The decrease in the size of silica-coated Fe_3_O_4_ NPs or Fe_3_O_4_@SiO_2_ (Figure 11(c)) is due to the capping effect of silicon dioxide. The as-synthesized magnetite dissolved in aqueous solution for the DLS measurement was strongly agglomerated as confirmed by the SEM image of Fe_3_O_4_ Tween-80 at 30°C materials after DLS measurement (Figure 12).

### 3.2. Antimicrobial Study

The antimicrobial study revealed that the microorganisms were sensitive to the test samples in varying magnitudes. According to the results obtained as presented in Table 1, the maximum inhibition zone was recorded for *P. aeruginosa* bacteria in 15 mg/mL concentrations of Fe_3_O_4_-30T and Fe_3_O_4_-30 SDS zone of inhibition (ZOI) was found to be 19 mm. It is followed by silica-coated Fe_3_O_4_ NPs, Fe_3_O_4_-50T, and Fe_3_O_4_-30T dilute as their zone of inhibition was found to be 15, 14, and 14 mm, respectively. Other samples showed moderate activity against *P. aeruginosa*. Fe_3_O_4_-30T, Fe_3_O_4_-30 SDS, and silica-coated Fe_3_O_4_ NPs also showed excellent activity against *E. coli* as their zone of inhibition was found to be 18 mm. It is followed by Fe_3_O_4_-50T and Fe_3_O_4_-30T solution as the zone of inhibition was observed to be 17 and 15 mm, respectively. Fe_3_O_4_-30 SDS, Fe_3_O_4_-30T conc., Fe_3_O_4_-30T, and Fe_3_O_4_-80 SDS NPs showed good activity against *B. subtilis* as their zone of inhibition was found to be 14, 14, 13, 13, and 13 mm, respectively. Silica-coated Fe_3_O_4_ and Fe_3_O_4_-30T showed fine activity against *S. aureus* with the zone of inhibition being 14 and 13 mm as compared with other samples. The results in Table 1 and Figure 12 show that generally Gram-positive bacteria are more resistant to Fe_3_O_4_ nanoparticles in comparison with Gram-negative bacteria related to their cell wall structure, cell physiology, metabolism, or degree of contact [32, 33]. Moreover, the diameter of the inhibition zone was different for the different types of bacteria [34].

## 4. Conclusion

The bare Fe_3_O_4_ and silica-coated Fe_3_O_4_nanoparticles were successfully synthesized via microemulsion method using Tween-80 and SDS surfactants by varying temperature and precursor concentration. Thus, the microemulsion method was found to be an effective method to get controllable size nanoparticles. Fourier transform infrared spectra and X-ray diffraction showed that the Fe_3_O_4_ NPs were successfully coated by silica. The SEM result showed that the morphology of Fe_3_O_4_ nanoparticles synthesized using Tween-80 is homogeneous and uniformly distributed with flower-like shape. Large average particle size was measured by DLS for bare/uncoated Fe_3_O_4_ particles because of agglomeration. The Fe_3_O_4_ NPs synthesized using Tween-80 showed better antimicrobial properties on both Gram-positive and Gram-negative bacterial strains and *Candida ablicans* fungi. Silica-coated Fe_3_O_4_ NPs also showed comparable antimicrobial activity with Fe_3_O_4_ NPs synthesized using Tween-80.

## Figures and Tables

**Table 1:** Zone of inhibition (mm) of Fe_3_O_4_ NPs and silica-coated NPs against Gram-negative and Gram-positive bacterial strains and *C. albicans* fungi.

Samples	Zone of inhibition (mm) (Diameter)									
	Gram-positive bacteria				Gram-negative bacteria				*C. albicans*(ATCC9955)	
	*Staphylococcus aurous*(ATCC 25923)		*Bacillus subtilis*(ATCC6633)		*Escherichia coli*(ATCC25922)		*Pseudomonas aeruginosa* (ATCC7553)			
	C_1_ 10 mg/mL	C_2_ 15 mg/mL	C_1_ 10 mg/mL	C_2_ 15 mg/mL	C_1_ 10 mg/mL	C_2_ 15 mg/mL	C_1_10 mg/mL	C_2_15 mg/mL	C_1_10 mg/mL	C_2_15 mg/mL
Fe_3_O_4_-30T	8	11	10	13	15	18	14	19	11	15
Fe_3_O_4_-50T	8	11	8	10	11	15	12	14	9	10
Fe_3_O_4_-80T	9	11	7	11	6	9	9	11	7	8
Fe_3_O_4_-30 SDS	8	10	9	14	7	11	14	19	12	13
Fe_3_O_4_-50 SDS	9	10	9	12	9	11	8	12	11	12
Fe_3_O_4_-80 SDS	8	10	9	13	13	18	10	12	9	12
Fe_3_O_4_-30T dil.	8	13	8	13	6	11	9	14	12	14
Fe_3_O_4_-30T conc.	7	9	7	14	14	17	10	12	11	12
Silica-coated Fe_3_O_4_	10	14	11	12	14	18	7	15	8	10
Gentamicin (+ve)	12	14	14	14	14					
DMSO (−)	No	No	No	No	No					

## Data Availability

The data used to support the findings of this study are available from the corresponding author upon request.
